# Expression and prognostic relevance of Cyclophilin A and matrix metalloproteinase 9 in esophageal squamous cell carcinoma

**DOI:** 10.1186/1746-1596-8-207

**Published:** 2013-12-18

**Authors:** Yi Li, Hui Guo, Danfeng Dong, Huili Wu, Enxiao Li

**Affiliations:** 1 Department of Oncology, First Affiliated Hospital, Medical School, Xi’an Jiaotong University, Yanta West Road No. 277, Xi’an 710061, Shaanxi Province, China

**Keywords:** Esophageal squamous cell carcinoma, Cyclophilin A, Matrix metalloproteinase 9

## Abstract

**Aims:**

To guide clinicians in selecting treatment options for esophageal squamous cell carcinoma (ESCC) patients, reliable markers predictive of clinical outcome are desirable. This study analyzed the correlation of cyclophilin A (CypA) and matrix metalloproteinase 9 (MMP9) in ESCC and their relationships to clinicopathological features and survival.

**Methods:**

We immunohistochemically investigated 70 specimens of ESCC tissues using CypA and MMP9 antibodies. Then, the correlations between CypA and MMP9 expression and clinicopathological features and its prognostic relevance were determined.

**Results:**

Significant correlations were only found in high level of CypA and MMP9 expression with tumor differentiation and lymph node status. Significant positive correlations were found between the expression status of CypA and that of MMP9. Overexpression of CypA and metastasis were significantly associated with shorter progression free survival times in univariate analysis. Multivariate analysis confirmed that CypA expression was an independent prognostic factor.

**Conclusions:**

CypA might be correlated with the differentiation, and its elevated expression may be an adverse prognostic indicator for the patients of ESCC. CypA/MMP9 signal pathway may be attributed to the malignant transformation of ESCC, and attention should be paid to a possible target for therapy.

**Virtual slides:**

The virtual slide(s) for this article can be found here: http://www.diagnosticpathology.diagnomx.eu/vs/1166551968105508.

## Introduction

Esophageal squamous cell carcinoma (ESCC) is a highly aggressive neoplasm with geographic characters and poor prognosis. About one-half of all ESCC cases in the world occur in China [1]. Despite a myriad of improvements in both diagnostic and therapeutic techniques over the past three decades, ESCC continues to have a poor prognosis, with 5-year survival rates between 10-13% [2]. Research over the last 30 years has identified a number of genetic alterations relating to induction of ESCC. Besides, some of them were shown to be of prognostic significance. However, further comprehensive investigations and new clues were expected. To guide clinicians in selecting treatment options for ESCC patients, reliable markers predictive of poor clinical outcome are desirable.

Cyclophilin A (CypA) was originally identified as the intracellular receptor for cyclosporin A (CsA) [3]. It is implicated in several diseases, including viral infection, cardiovascular disease, inflammatory diseases, and cancer [4-7]. The role of CypA in cancer has recently drawn attention. Various cancers, including ESCC over-expressed CypA [8-13]. Although much effort has been devoted to the function of CypA in cancer, but few research has been undertaken to evaluate the clinical value of CypA in ESCC. Matrix metalloproteinases (MMPs) are a highly regulated super family of zincdependent endopeptidases causally associated with the development and progression of tumors [14]. MMP9, a target gene of CypA, was revealed over-expression in ESCC [15].

In this study, we investigated whether expression levels of CypA and MMP9 have prognostic significance in ESCC. Immunohistochemical expression of CypA and MMP9 were examined in a total of 70 ESCC patients who underwent a surgical resection without any neoadjuvant treatment. We also investigated whether the expression levels of CypA correlate with that of MMP9 in this patient population and their prognostic value.

## Materials and methods

### Patients

ESCC patients who were confirmed by pathology were collected in the First Affiliated Hospital of Xi’an Jiaotong University from 2004 to 2009, and also received surgical treatment. After following-up visits, 70 patients who had complete clinical data were selected. None of these 70 patients received neoadjuvant therapy before operation. Patients were followed closely until December 31, 2012, and the range of the follow-up period was 1 to 25 months (median, 9.33 months). Computed tomography (CT) was performed at least every 6 months to detect recurrence. Differentiation grade, TNM stage and lymph node status were conducted according to UICC/AJCC TNM classification (seventh edition). The clinicopathological features of patients are shown in Table 1. The Institutional Ethics Committee approval for this project was obtained from Institutional Review Board of First Affiliated Hospital of Xi’an Jiaotong University.

**Table 1:** Clinicopathologic variables and the expression status of CypA

**Variables**	**N**	**CypA**		***P***
		**Low**	**High**	
Age				0.242
<65	48	13	35	
≥65	22	3	19	
Gender				0.555
Male	45	9	36	
Female	25	7	18	
Smoking				0.343
Yes (>40 pack-years)	51	10	41	
No	19	6	13	
Drink				0.580
Yes (>50 ml/day)	39	10	29	
No	31	6	25	
Differentiation				<0.01
Well + Moderate	39	16	23	
Poor	31	0	31	
TNM stage				0.123
I–II	22	8	14	
III–IV	48	8	40	
Lymph node status				0.010
Metastasis	38	4	34	
No metastasis	32	12	20	

### Immunohistochemical staining

Tissue specimens were fixed in neutral buffered formalin (10% v/v formalin in water; pH 7.4) and embedded in paraffin wax. Serial sections of 4-μm thickness were cut and mounted on charged glass slides. The monoclonal antibody against CypA (1:400; Abcam, Cambridge, UK) and MMP9 (1:800; Santa Cruz Biotechnology, CA, USA) were used respectively. The Streptavidin-Peroxidase technique (Golden Bridge International: SP-9000) was used. An irrelevant rabbit antiserum served as a negative control. Sections were counterstained with Mayer’s hematoxylin.

### Immunohistochemical analysis

Two pathologists who were blinded to clinical evaluated staining results independently and co-observed for a consensus when they were divergent with the method as described. Both of the percentage of positive cells and the strength of the staining were considered in the following method. 5 degree magnification visions were chose randomly under the optical microscope, the calculation of results as followed: the percentage of positive cells in 0%-5% was counted 0; the percentage of positive cells in 5%-25% was counted 1; 26%-50% was counted 2; 51%-75% was counted 3; ≥ 76% was counted 4. On the respect of staining strength, the score for tumor cells without stain is 0; straw yellow for 1; brown for 2; tan for 3. The staining index score was the sum of the items above. For the purpose of statistical analysis, the median of this series (25% of malignant cells showing a stronger intensity than adjacent non-tumor epithelium) was used as a cutoff value to distinguish tumors with a low (<25) or high (≥25%) level of CypA and MMP9 expression.

### Statistical analysis

Statistical analysis was done using the SPSS software package (version 13.0, SPSS Institute). The association between staining index and other categorical factors potentially predictive of prognosis was analyzed using the Fisher’s exact test. The Spearman’s rank correlation coefficient was used for analyzing the association of MMP9 expression levels with CypA expression status. Progression-free survival (PFS) was defined as the time from the first day of treatment to the time of disease progression. Survival curve and median survival were estimated by the Kaplan-Meier method. Their differences were verified by log-rank test. Multivariate analysis was done using the Cox proportional hazard regression analysis. Results were considered statistically significant if P < 0.05.

## Results

### Expression of CypA and MMP9 in ESCC and their relationships to clinicopathological variables

**Figure 1:**
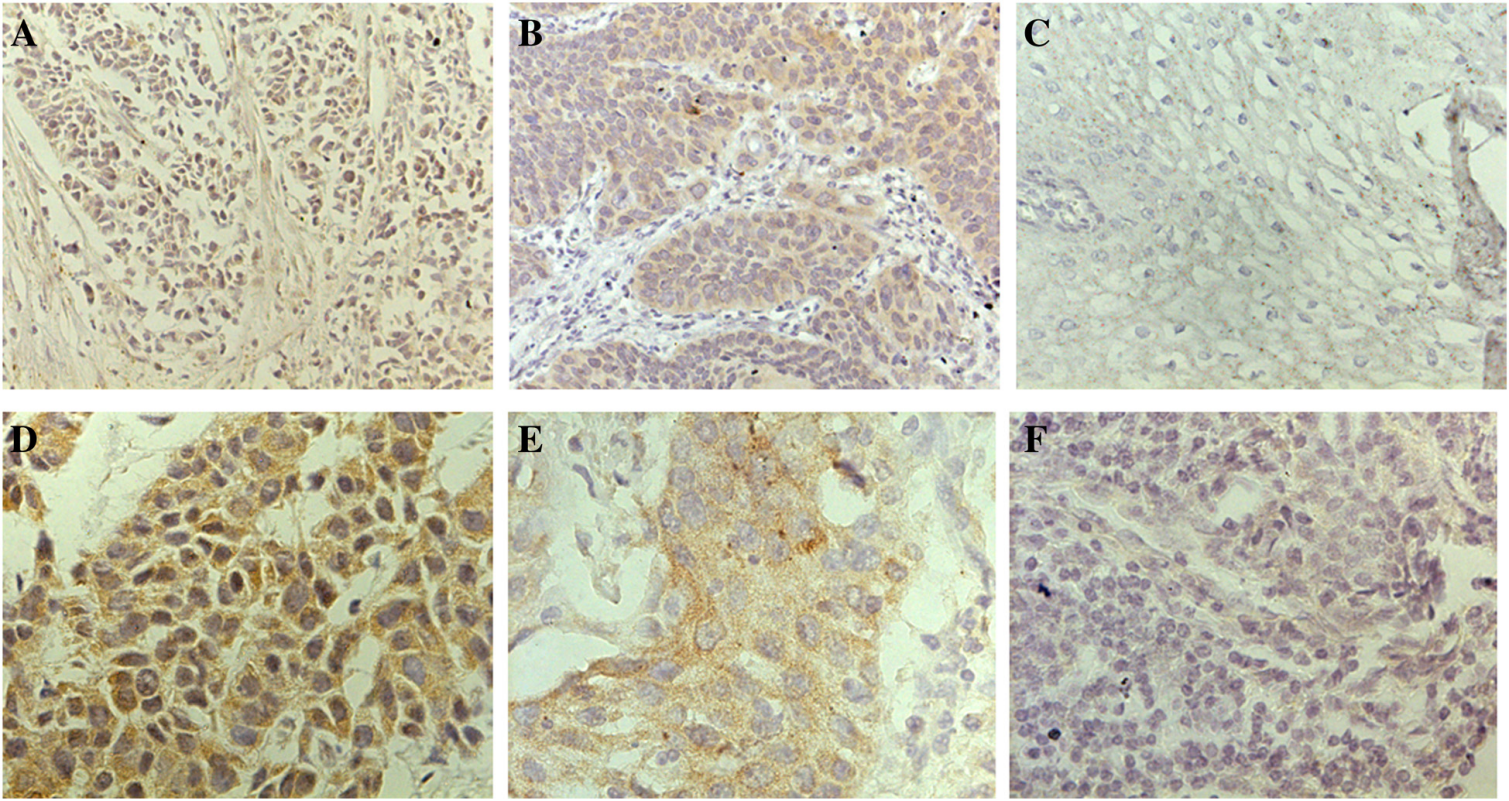
**Immunohistochemical expression of CypA and MMP9 in esophageal squamous cell carcinoma. A**, **D** Typical immunohistological features with high levels of CypA expression in esophageal squamous cell carcinoma (ESCC). The CypA staining shown nuclear and cytoplasmic localization; **B**, **E** Typical immunohistological features with high levels of MMP9 in ESCC. The MMP9 staining was present in the cytoplasm of tumor cells; **C**, **F** Negative staining in ESCC. Magnifications: **A-C** × 200, **D-F** × 400.

Levels of CypA and MMP9 were evaluated by immunohistochemical analysis. CypA immunoreactivity showed nuclear and cytoplasmic localization, while MMP9 was found primarily in the cytosol. Figure 1 shows representative expression patterns of CypA and MMP9 in ESCC. Both high level of CypA and MMP9 expression significantly correlated with the tumor differentiation and metastasis. However, the high level rates were not significantly correlated with gender, age, drink, smoking, and TNM stage (Tables 1 and 2).

**Table 2:** Clinicopathologic variables and the expression status of MMP9

**Variables**	**N**	**MMP9**		***P***
		**Low**	**High**	
Age				1.000
<65	48	14	34	
≥65	22	6	16	
Gender				0.783
Male	45	12	33	
Female	25	8	17	
Smoking			0.146	0.301
Yes (>40 pack-years)	51	12	39	
No	19	8	11	
Drink				0.427
Yes (>50 ml/day)	39	13	26	
No	31	7	24	
Differentiation				0.003
Well + Moderate	39	17	22	
Poor	31	3	28	
TNM stage				0.397
I–II	22	8	12	
III–IV	48	14	36	
Lymph node status				0.016
Metastasis	38	6	32	
No metastasis	32	14	18	

### Association of CypA expression levels with MMP9 expression status

Since CypA is one of the important transcription factors for MMP9 gene expression, we next investigated the association of CypA expression levels with MMP9 expression status (Table 3). Of the 54 tumors containing a high level of CypA immunoreactivity, a total of 50 cases displayed a high level of MMP9 expression. We calculated the Spearman’s rank correlation coefficient to evaluate the linear relationship. There was statistically significant association of CypA expression status with MMP9 expression levels (r = 0.861,P < 0.01).

**Table 3:** Association of MMP9 expression levels with CypA expression status

**Variables**	**Total**	**MMP9**		**P**	**r**
		**Low**	**High**		
CypA				<0.01	0.861
Low	16	16	0		
High	54	4	50		

### Survival analysis

**Figure 2:**
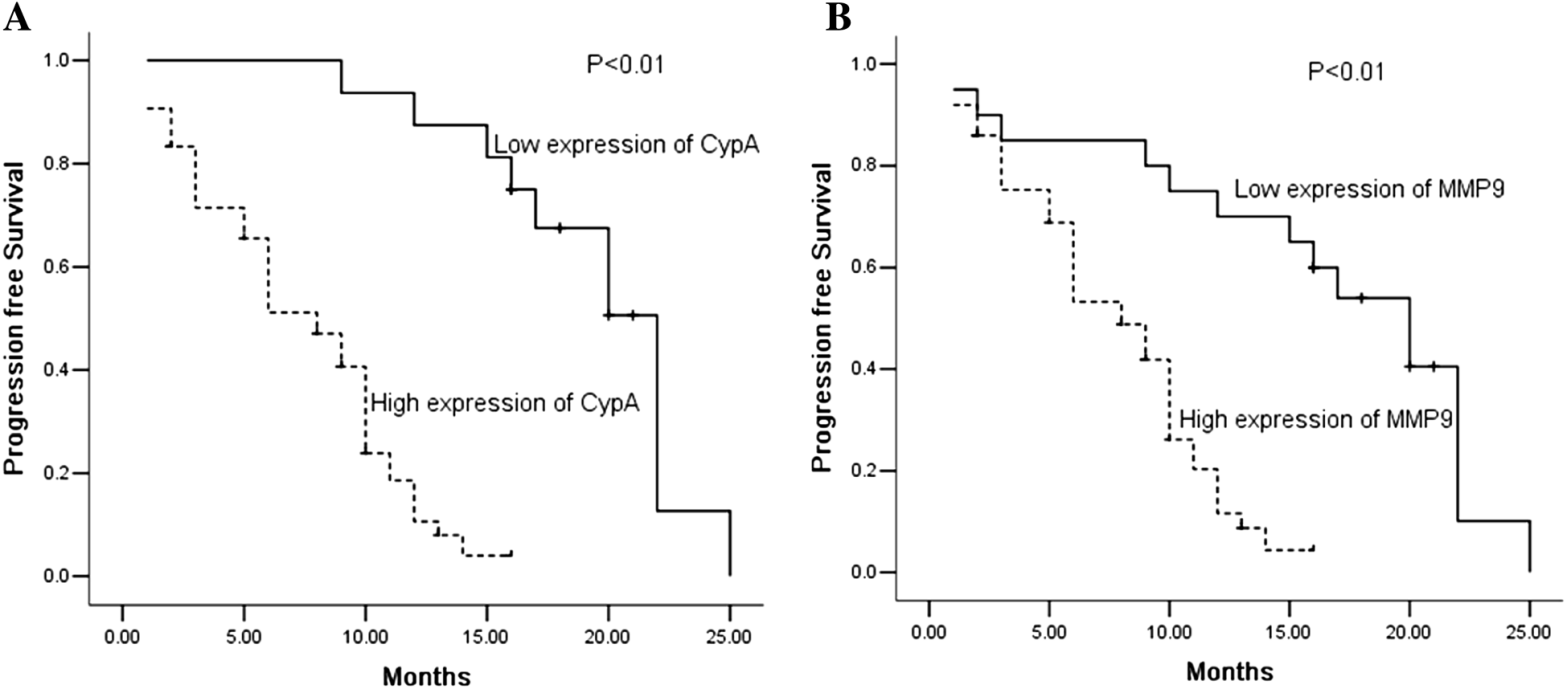
**Kaplan-Meier survival analysis.** Cumulative progression free survival differences between patients with high and low levels of protein expression. P value was obtained using the log-rank test of the difference. **A** CypA; **B** MMP9.

Kaplan-Meier analysis was used to calculate the impact of classic clinicopathologic features and protein expression on PFS (Table 4, Figure 2). CypA, MMP9, differentiation, and metastasis were associated with decreased survival (P < 0.05), whereas other clinicopathological variables were not significant. Cox regression analysis revealed a statistically significant correlation between metastasis and CypA expression and PFS (P < 0.01, Table 5).

**Table 4:** Univariate analysis for progression free survival

**Variables**	**N**	**Progression free survival (months)**		***P***
		**Median ± SE**	**95% CI**	
CypA				<0.01
Low	16	16.42 ± 1.16	17.08-21.76	
High	54	7.42 ± 0.60	6.24-8.59	
MMP9				<0.01
Low	20	16.332 ± 1.75	10.91-14.93	
High	50	7.70 ± 0.62	6.49-8.91	
Age				0.220
<65	48	11.47 ± 1.22	9.08-13.87	
≥65	22	9.44 ± 1.23	7.03-11.84	
Gender				0.950
Male	45	10.82 ± 1.24	8.38-13.25	
Female	25	11.30 ± 1.45	8.46-14.15	
Smoking				0.269
Yes (>40 pack-years)	51	10.06 ± 1.02	8.01-12.05	
No	19	12.35 ± 1.82	8.79-15.92	
Drink				0.406
Yes (>50 ml/day)	39	11.28 ± 1.25	8.83-13.72	
No	31	10.07 ± 11.32	7.48-12.66	
Differentiation				<0.01
Well + Moderate	39	13.33 ± 1.28	10.82-15.84	
Poor	31	7.07 ± 0.78	5.54-8.60	
TNM stage				0.295
I–II	22	12.02 ± 1.87	8.36-15.67	
III–IV	48	10.20 ± 1.02	8.20-12.19	
Lymph node				0.041
Metastasis	38	8.79 ± 1.20	6.44-11.14	
No metastasis	32	12.75 ± 1.20	10.40-15.10	

**Table 5:** Multivariate Cox proportional hazards analysis for progression free survival

**Variables**	**Progression free survival**		***P***
	**HR**	**95% CI**	
CypA			
Low vs High	26.22	4.46-154.02	<0.01
MMP9			
Low vs High	2.945	0.94-9.24	0.064
Lymph node			
No metastasis vs Metastasis	0.587	0.271-1.274	0.178
TNM stage			
I–II vs III–IV	0.657	0.319-1.353	0.255
Differentiation			
Poor vs Well + Moderate	1.939	0.819-4.59	0.132

## Discussion

CypA, an 18-kDa cytosolic protein that is ubiquitously expressed in prokaryotes and eukaryotes, is an important component in protein folding. CypA has an activity of peptidylprolyl cis-trans isomerase, which may play important roles in protein folding, trafficking, assembly, immune-modulator and cell signaling. It displays an unusually high expression in several cancer types and correlates with poor outcome of the patients. Overexpression of CypA was first demonstrated in hepatocellular carcinoma in 1998 [16], then a growing number of reports focus on the role of CypA in cancer. Different types of cancers, including lung cancer, colorectal cancer, pancreatic cancer, breast cancer, squamous cell carcinoma, and melanoma exhibit upregulated CypA [8-13]. Some researchers have investigated the function of CypA during tumor progression, including the stimulation of proliferation, blockade of apoptosis, regulation of metastasis, malignant transformation and drug resistant [17]. In the study of Qi et al. [12], CypA was differentially expressed between esophageal cancer cell lines and immortal cell line, which suggested that CypA may implicated in the esophageal malignant transformation processes. Even so, the expression and significance of CypA in ESCC remains incompletely understood.

Metastasis is the primary cause of morbidity and mortality in cancer patients. Stable CypA RNA-interfered breast cancer and osteosarcoma cells showed reduced migratory capacity [18]. MMPs were also associated with tumor invasion and migration [19]. MMP9 plays a pivotal role in the degradation of ECM [20]. In a microarray results, MMP9 were found regulated by CypA. RNA interference assay also demonstrates that MMP9 were regulated by CypA in SKHep1 cells [21]. The same result was found by Qian et al. in non-small cell lung cancer [22]. Further more, increased expression of MMP9 was found in ESCC [15].

In this study, we showed that CypA and MMP9 were highly expressed in ESCC. Both high level of CypA and MMP9 expression significantly correlated with the tumor differentiation and metastasis. So, we may conclude that both CypA and MMP9 have an important role in the progression of ESCC. Next, significant positive correlations (Spearmen rank correlation) were found between the expression status of CypA and that of MMP9. This means MMP9 may be one of CypA related interacting partners, suggesting that CypA may regulate the expression of MMP9 . However, the exact molecular mechanisms remain to be clarified. Overall, the available data so far suggest that CypA pathway may well be related to the genetic changes implicated in ESCC progression.

There are lots of reports about different risk factors in ESCC, including NDRG2, HSPA2, HAX-1, USP9X, and so on [23-27]. The prognostic value of MMP-9 in cancer was also investigated, but there were few reports about that of CypA [28-31]. Although it was found no prognostic significance of CypA in non-small cell lung cancer, but overexpression of CypA was associated with decreased survival in various cancers, including endometrial carcinoma, tongue squamous cell carcinoma, and renal cell carcinoma. However, the prognosis value of CypA in ESCC remains unknown. According to our results, MMP-9 failed to predict patients’ prognosis, whereas CypA was shown to be an independent prognostic indicator in patients with ESCC. Take all these results into consideration, CypA might be available not only as clinical predictors, but also as targets for ESCC treatment. We will focus on both the prognostic and treatment value of CypA in furture.

The current study suggested that the high expression of CypA proteins was associated with important clinicopathological parameters in ESCC. There was a significant positive correlation between the expression status of CypA and that of MMP9. Further, CypA was an important prognostic indicator in cases of ESCC. Therefore, CypA/MMP9 signal pathway may be attributed to the malignant transformation of ESCC, and attention should be paid to a possible target for therapy.

## Competing interests

The authors declare that they have no competing interests.

## Authors’ contributions

YL and HG constructed the manuscript. YL, HG and DD carried out immunohistochemical study. DD, HW and EL were responsible for clinical data; evaluated clinical data; formed analysis of relation between clinical data and survival data. All authors read and approval the final manuscript.

## Authors’ information

Yi Li, Hui Guo are co-first authors.
